# Prognostic Value of Pentraxin-3 Level in Patients with STEMI and Its Relationship with Heart Failure and Markers of Oxidative Stress

**DOI:** 10.1155/2015/159051

**Published:** 2015-04-01

**Authors:** Marie Tomandlova, Jiri Jarkovsky, Josef Tomandl, Lenka Kubkova, Petr Kala, Simona Littnerova, Jana Gottwaldova, Petr Kubena, Eva Ganovska, Martin Poloczek, Jindrich Spinar, Christian Mueller, Alexandre Mebazaa, Monika Pavkova Goldbergova, Jiri Parenica

**Affiliations:** ^1^Department of Biochemistry, Faculty of Medicine, Masaryk University, 625 00 Brno, Czech Republic; ^2^Institute of Biostatistics and Analyses, Masaryk University, 625 00 Brno, Czech Republic; ^3^Department of Cardiovascular Disease, International Clinical Research Center, St. Anne's University Hospital, 656 91 Brno, Czech Republic; ^4^Department of Cardiology, University Hospital Brno, 625 00 Brno, Czech Republic; ^5^Faculty of Medicine, Masaryk University, 625 00 Brno, Czech Republic; ^6^Department of Biochemistry, University Hospital Brno, 625 00 Brno, Czech Republic; ^7^Department of Internal Medicine, University Hospital, 4031 Basel, Switzerland; ^8^Department of Anesthesiology and Critical Care Medicine, Saint Luis Lariboisière University Hospital, 75475 Paris, France; ^9^Cardiac Diseases and Biomarkers, INSERM UMR 942, Saint Luis Lariboisière University Hospital, 75475 Paris, France; ^10^Institute of Pathological Physiology, Faculty of Medicine, Masaryk University, 625 00 Brno, Czech Republic

## Abstract

*Objective*. Pentraxin-3 (PTX3) appears to have a cardioprotective effect through a positive influence against postreperfusion damage. This study assesses the prognostic value of PTX3 level and its relationship with clinical parameters and markers of oxidative stress and nitric oxide metabolism in patients with ST-elevation myocardial infarction (STEMI). *Methods*. Plasma/serum levels of several biomarkers of inflammation and oxidative stress and nitrite/nitrate were assessed upon admission and 24 h after STEMI onset in patients treated by primary percutaneous coronary intervention. *Results*. ROC analysis showed that plasma PTX3 at 24 h was a strong predictor of 30-day and 1-year mortality and independent predictor of combined end-point of left ventricle dysfunction or mortality in 1 year. The inflammatory response expressed by PTX3 had a significant relationship with age, heart failure, infarct size, impaired flow in the infarct-related artery, and renal function and positively correlated with neopterin, TNF-*α*, 8-hydroxy-2′-deoxyguanosine, and nitrite/nitrate. *Conclusions*. Plasma PTX3 at 24 h after STEMI onset is a strong predictor of 30-day and 1-year mortality. PTX3 as a single biomarker is comparable with currently used scoring systems (TIMI or GRACE) or B-type natriuretic peptide. PTX3 is also an independent predictor of combined end-point of left ventricle dysfunction or mortality in 1 year.

## 1. Introduction

Inflammation has a crucial role during the acute phase and healing process after acute myocardial infarction (MI). The inflammatory response is associated with the production of many mediators. Increased levels of C-reactive protein (CRP) during the MI have been demonstrated to be an independent negative prognostic factor [1, 2]. CRP activates complement via the classical pathway and thereby contributes to the lysis and removal of damaged myocardial cells [3]. An excessively high inflammatory response as expressed by overproduction of CRP may lead to worsening function and remodeling of the left ventricle [4, 5].

Pentraxin-3 (PTX3), also known as “long pentraxin,” belongs to the evolutionarily conserved pentraxin family, together with CRP. PTX3 seems to have, in comparison with CRP, a different pathophysiological role in patients with MI. PTX3 can bind to component C1q and thus prevent unwanted excessive activation of complement [6]. Binding of PTX3 to P-selectin reduces further neutrophil infiltration into the site of myocardial injury [7], and binding to activated platelets reduces proinflammatory and prothrombotic effects in MI patients [8]. Significantly greater myocardial damage has been demonstrated in* ptx3*-deficient mice compared with wild-type controls. The protective effect of PTX3 is probably caused by influencing of the postreperfusion damage of myocardial tissue [9].

PTX3 levels in plasma of the patients with MI increase rapidly and reach their peak 6–8 h from symptom onset [10]. Early increase in the plasma concentration of PTX3 in MI patients is associated with neutrophil degranulation [8]. Local production of PTX3 by different cells in affected areas contributes to increased plasma PTX3 concentrations in the later stages of MI [9, 11]. Mononuclear phagocytes, dendritic cells, fibroblasts, and vascular endothelial cells produce PTX3 in response to primary inflammatory signals, which are mainly from cytokines such as interleukin-1*β* and tumor necrosis factor alpha (TNF-*α*) [12]. Expression of PTX3 in a model of MI and reperfusion is controlled through nuclear factor-kappa B [13].

Increased PTX3 levels in ST-elevation myocardial infarction (STEMI) patients upon hospital admission have been demonstrated to be independent predictors of 3-month mortality, but not of readmission to hospital for acute heart failure (AHF) [10, 14]. A predictive value in non-ST-elevation myocardial infarction (NSTEMI) patients was not proven [15]. The current risk stratification in STEMI patients based only on basic clinical parameters is limited [16–18].

In the present study, we evaluated the 30-day and one-year prognostic value of PTX3 level in addition to a validated clinical model in consecutive STEMI patients treated by primary percutaneous coronary intervention (PCI), the one-year prognostic value of PTX3 level in addition to clinical model to predict combined end-point of mortality or left ventricle dysfunction, the relationship between PTX3 level and clinical parameters, and the association of plasma PTX3 level with other inflammatory markers, markers of oxidative stress, and markers associated with the metabolism of nitric oxide (NO).

## 2. Methods

### 2.1. Study Population

The study cohort was 262 patients with STEMI referred for primary PCI and admitted to the Coronary Care Unit of the Internal Cardiology Department of University Hospital Brno (Brno, Czech Republic). Exclusion criteria were age >80 years, known or newly diagnosed malignancy, inflammatory or connective-tissue disease, disease other than cardiovascular disease that would clearly limit the 1-year prognosis, and noncompliance with treatment. The study protocol complied with the Declaration of Helsinki and was approved by the Ethics Committees of the Faculty Hospital Brno. Written informed consent was obtained from all subjects before participation in the trial. The diagnosis of STEMI was made based on MI symptoms, appropriate electrocardiographic changes, and elevation of levels of troponin I. Time from onset of chest pain to primary PCI was <12 h. Left ventricular end-diastolic pressure (LVEDP) was measured before left ventricular angiography using a fluid-filled catheter (5-F pigtail catheter) with the patient in the supine position before primary PCI. Thrombolysis in myocardial infarction (TIMI) flow grade was assessed before and after primary PCI.

Patients were monitored prospectively in the cardiology outpatient department of University Hospital Brno. One-year mortality was assessed in all patients.

### 2.2. Echocardiography Assessment

Echocardiography was carried out during the index admission (between the third and fifth day after MI onset) and after one year. Left ventricular end-systolic volume (LVESV), left ventricular end-diastolic volume (LVEDV), and left ventricular ejection fraction (LVEF) were estimated using the biplanar Simpson's rule from apical two- and four-chamber views. Echocardiography was assessed by two operators using Vivid 7 or Vivid i (GE Vingmed Ultrasound).

### 2.3. Laboratory Methods

Venous blood samples were drawn immediately upon hospital admission (median of 3 h from onset of chest pain) and 24 h after onset of MI. Serum and EDTA-plasma samples were aliquoted and stored immediately at −80°C until assay. Standard biochemical and hematological blood tests were done upon hospital admission while other tests were done within one to four months of collection.

Plasma levels of troponin I and B-type natriuretic peptide (BNP) were analyzed using the microparticle enzyme immunoassays (AxSYM, Abbott Laboratories, IL, USA). Plasma levels of PTX3 were measured with an enzyme-linked immunosorbent assay (ELISA) (R&D Systems, MN, USA) and plasma levels of TNF-*α* with an ELISA (Invitrogen, CA, USA). Serum concentrations of neopterin were measured with an ELISA (IBL International, Germany), serum concentrations of 8-hydroxy-2′-deoxyguanosine (8-OHdG) with an ELISA (Cayman Chemicals, MI, USA), and serum concentrations of superoxide dismutase (SOD) with an ELISA kit (Bender MedSystems, Austria). Plasma levels of NO were evaluated by measuring the nitrite/nitrate (NO_*x*_) using a colorimetric assay kit based on the Griess reaction (R&D Systems). Ferric-reducing ability of plasma (FRAP) was measured using the method of Benzie and Strain [19] with modifications on the assay on 96-well microplate on a Spectramax 340PC Microplate Reader (Molecular Devices, CA, USA). The plasma level of malondialdehyde (MDA) was assessed by high-performance liquid chromatography (HPLC) with fluorescent detection (Shimadzu 10A series HPLC system, Shimadzu Corp., Japan) after derivation by thiobarbituric acid as described by Khoschsorur et al. [20]. Plasma levels of total low-molecular-weight thiols were measured by HPLC with fluorescent detection (Shimadzu) as described by Vester and Rasmussen [21]. The plasma level of vitamin A and vitamin E was measured after extraction by HPLC with UV detection (Shimadzu) [22].

### 2.4. Statistical Methods

Standard descriptive statistics were applied in the analysis: absolute and relative frequencies for categorical variables and median supplemented by 5th–95th percentile ranges for quantitative variables. The significance of differences among groups was analyzed using the maximum-likelihood chi-square test for categorical variables and Mann-Whitney or Kruskal-Wallis tests for quantitative variables. The relationship between quantitative variables was assessed using the Spearman correlation coefficient. ROC analyses were adopted for the description of the predictive power of variables and identification of optimal cut-off points using Youden's J statistic. Reclassification analyses (net reclassification improvement (NRI), integrated discrimination improvement (IDI), and category-free net reclassification improvement (cfNRI)) were used for the characterization of improvement of standard TIMI and GRACE risk scores by the addition of new markers and for the comparison of multivariate models with and without PTX3. Analyses were computed using SPSS 22.0.0.1 (IBM Corporation, 2014) and R version 3.1.2 (The R Foundation for Statistical Computing, 2014) with library PredictABEL [23]. The level of significance was set at *α* = 0.05.

## 3. Results

The comparison of PTX3 levels upon admission and 24 h after onset of MI demonstrated a significant rise of values (0.96 (0.24–4.02) versus 2.13 (0.49–21.64) ng/mL; *P* < 0.001). As the main aim of the study was to evaluate a prognostic value of PTX3, we compared the values of area under the curve (AUC) of ROC analysis for both time points. We found that PTX3 after 24 h had a significantly higher prognostic value than PTX3 upon admission (see below). Based on this result, all following statistical analyses were done with PTX3 after 24 h.

The baseline characteristics of the study cohort divided into tertiles according to plasma PTX3 levels after 24 h are presented in Table 1. Increasing tertiles of PTX3 were associated with older age, diabetes mellitus, increasing LVEDP, and decreased LVEF as shown in Table 1. Moreover, patients with higher values of PTX3 (2nd and 3rd tertiles) had after reperfusion by primary PCI final TIMI flow in the infarct-related artery more often lower than level 3 in comparison with patients from the 1st tertile. Patients within the highest tertile of PTX3 level had more often signs of AHF and acute kidney injury.

Table 2 presents the values of biomarkers evaluated within tertiles of PTX3 after 24 h. Increased values of PTX3 were accompanied by increasing levels of troponin I, BNP, NO_*x*_, TNF-*α*, neopterin, SOD, and FRAP. In addition, the values of some laboratory parameters upon admission within tertiles of PTX3 at 24 h are presented in Table 3.

Comparison of median values of PTX3 in selected groups of patients is shown in Table 4. Significantly higher PTX3 levels were noted in patients with AHF, acute kidney injury, diabetes mellitus, and nonhypercholesterolemic patients. It seems that higher PTX3 levels were slightly associated with final TIMI flow values of 0 and 1 (i.e., those with an unsuccessful reperfusion above all on microcirculatory level). This association has not been shown to be statistically significant.

The relationship between PTX3 level at 24 h and clinical/biochemical parameters as expressed by the Spearman correlation coefficient is shown in Table 5. Univariate analysis revealed the most powerful association of PTX3 level with level of BNP, neopterin, troponin I, NO_*x*_, and TNF-*α* (all at 24 h) and, moreover, with level of BNP, 8-OHdG, glucose, and creatinine upon hospital admission. A negative correlation between PTX3 levels was found with LVEF.

**Figure 1:**
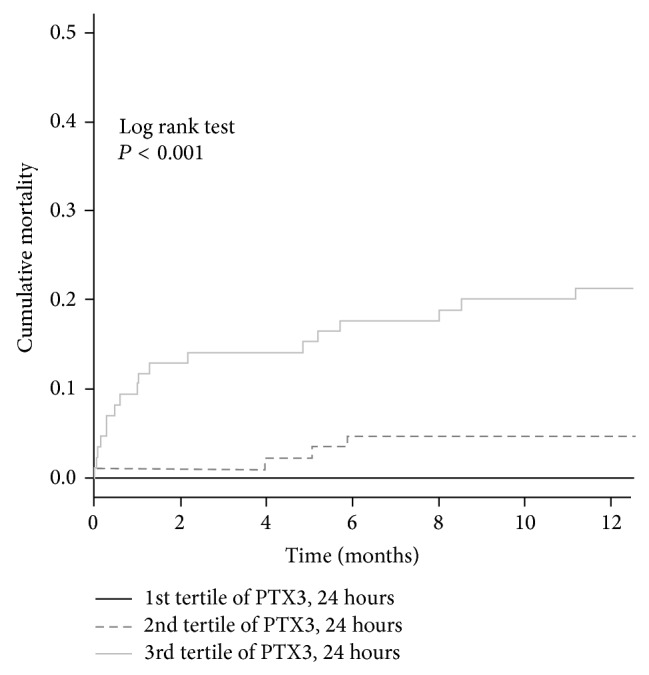
The Kaplan-Meier curves of 1-year mortality according to PTX3 level at 24 h after the onset of myocardial infarction.

Thirty-day mortality was 0% within the first tertile of PTX3, 1.1% within the second, and 10.5% within the third. One-year mortality was 0% within the first tertile, 4.5% within the second, and 20.9% within the third (Figure 1). We have found a high predictive value of PTX3 level at 24 h for prediction of 30-day and 1-year mortality (AUC 0.873 and 0.835, resp.; both *P* < 0.001; sensitivity 100% and specificity 66%) with optimal cut-off value > 3.07 ng/mL. According to the C-statistics, AUC of the previously validated scoring system TIMI for 30-day and 1-year mortality was 0.795 and 0.691 (both *P* < 0.001) for our dataset, respectively, and AUC of GRACE risk score for 30-day and 1-year mortality was 0.841 and 0.783 (both *P* < 0.001), respectively, as shown in Table 6. Also we found a high predictive value of BNP level at 24 h for 30-day and 1-year mortality (both AUC 0.812; *P* < 0.001). Addition of PTX3 level at 24 h to TIMI and GRACE risk scores led to nonsignificant improvement of a prognostic value of the clinical model for prediction of 30-day and 1-year mortality (Table 6). PTX3 upon admission demonstrated significant but moderate prognostic value for prediction of 1-year mortality but not for 30-day mortality. Addition of PTX3 upon admission to TIMI risk score led to improvement of prognostic value as evaluated by the AUC of the C-statistics. Also, addition of PTX3 upon admission to GRACE score led to improvement of prognostic value of GRACE score as evaluated by the AUC of the ROC analyses (Table 6), but there was no significant improvement in risk reclassification according to NRI or IDI, and there was no significant improvement for prediction of 1-year mortality.

There was also an evaluation of the effect of PTX3 levels on the combined end-point of left ventricular dysfunction and mortality at one year. Results of C-statistics (Table 7) demonstrate the predictive ability of PTX3. For comparison, the two most commonly used biomarkers in patients with acute coronary syndrome are also shown, that is, troponin I and BNP.

Moreover, a multivariate model including basic clinical parameters, troponin I and BNP, is presented in Table 8. The maximum troponin levels reveal the extent of myocardial necrosis, levels of BNP indicate left ventricular dysfunction. Independent prognostic factors in a multivariate model were, in addition, heart rate and disabilities of all three coronary arteries. According to C-statistics, AUC was 0.866 (95% CI 0.817–0.916; *P* < 0.001). Addition of PTX3 level at 24 h to the previous model led to an increase of AUC to 0.878 (95% CI 0.830–0.925; *P* < 0.001). Reclassification analysis using cfNR and IDI demonstrated that PTX3 led to a significant improvement in the clinical model including BNP and troponin I (Table 8).

## 4. Discussion

In this study, we reported that PTX3 level determined upon hospital admission (≈3 h after MI onset) was significantly lower compared with that determined 24 h after MI onset. Also, we reported that PTX3 level 24 h after MI onset had greater prognostic value compared with the value upon hospital admission. Setting the time of sampling (24 h after the onset of chest pain) is close to the expected significant rise of PTX3 levels due to MI [9–11]. Moreover, the time of sampling is exactly associated with MI onset and it is the same for all patients.

Increased values of PTX3 were related to several factors: age; AHF and left ventricular overload expressed by several parameters (increased LVEDP, Killip heart failure class, and increased BNP level); extent of infarct size (as expressed by level of troponin I); renal function (in particular its worsening according to the number of patients with acute kidney injury); and impaired flow of infarct-related arteries (as expressed by TIMI flow before and after PCI). Furthermore, the level of PTX3 was slightly correlated with other markers of inflammation (neopterin and TNF-*α*), marker of nitric oxide synthase activity (NO_*x*_), and markers associated with oxidative stress (SOD, FRAP, and 8-OHdG upon admission).

We found a PTX3 level at 24 h to be a strong predictor of 30-day and 1-year mortality at least as good as previously validated clinical risk scores (GRACE and TIMI) or BNP level at 24 h.

The actual values of PTX3 and BNP upon hospital admission were not significant predictors of short-term mortality and had only a moderate prognostic value for 1-year mortality. An increase of a prognostic value of TIMI and GRACE risk scores after addition of PTX3 upon hospital admission was not also significant.

For the first time, according to our knowledge, we evaluated the effect of PTX3 levels on the development of a combined end-point of left ventricular dysfunction (EF < 40%) or mortality at one year. Since the combined end-point is not a validated clinical model, we have developed a multivariate model, in which, among others, we involved also the most commonly used biomarkers of acute coronary syndrome, that is, troponin I and BNP. PTX3 added to this model was evaluated as an independent predictor, which, moreover, led to a significant improvement in risk reclassification of patients.

One important factor in the evaluation of biomarkers in MI is setting the time of measurement of biomarkers in a relation to the onset of chest pain. In our opinion it is preferable to specify the time of measurement of biomarkers according to the time from onset of chest pain over the time of admission to the critical care unit. Sampling of PTX3 at two fixed predefined times, upon admission and 24 h after the onset of chest pain, can be considered to be an advantage in comparison with those chosen in previous studies [10, 14].

Our results are consistent with previous works. Increased production of PTX3 due to heart failure and increased physical stress (in our case expressed by the LVEDP value) has been described by Peri et al. [10] when patients with MI had higher PTX3 levels compared with healthy controls. Moreover, a correlation between CRP and PTX3 levels was not found in this group of patients. Latini et al. [14] demonstrated that, in patients with STEMI treated by thrombolysis, the PTX3 level was associated with heart failure and was confirmed as an independent prognostic factor of 3-month mortality. In addition, they found a very weak correlation between PTX3 and CRP levels.

It is known that production of PTX3 may also be influenced by genetic variants. Three common PTX3 polymorphisms (rs2305619, rs3816257, and rs1840680) have been demonstrated to be factors influencing PTX3 levels in patients with MI [24]. Unfortunately, based on performed tests, we could not assess if individuals with higher levels of PTX3 as a result of genetic predisposition had a better prognosis.

Moreover, PTX3 contributes to an inflammatory reaction at the site of infarction and probably should have a cardioprotective effect related to the modulation of activity of dendritic cells and decoupling of the C1q components of complement in the fluid phase [6, 9]. Complement activation alone in MI amplifies damage to the myocardium [25]. Our findings are not in compliance with findings from previous research that supposed protective effect of PTX3. According to our results, increasing concentrations of PTX3 are associated with poor prognosis of patient, that is, with higher risk of mortality and with development of left ventricular dysfunction. Whether the potential prognostic value of PTX3 reflects its influence on coagulation and complement activation or if high levels of PTX3 are a protective response reflecting the degree of inflammation and myocardial injury is not fully understood [26].

Our data support previous findings that an extensive inflammatory response plays an important adverse role in acute phase of MI [27, 28]. Recently published work in patients with NSTEMI demonstrated that inhibition of the p38 mitogen-activated protein kinase (p38MAPK) (a nexus point in inflammation, sensing and stimulating cytokine production, and driving cell migration and death) by new drug losmapimod decreases inflammatory response expressed by high sensitivity CRP at 72 h. At the same time the treatment led to significant decrease of BNP at 12 weeks [29]. Inflammatory reactions at the infarct site are associated with increased production of neopterin by macrophages after stimulation by interferon-*γ* [30]. Higher levels of neopterin are associated with the development of left ventricular dysfunction [31] and left ventricular remodelling [32] in patients with STEMI. TNF-*α* is rapidly released from resident mast cells and macrophages during MI [33] and its level is correlated closely with the extent of MI [34]. Our results showed a significant association between tertiles of PTX3 levels and those of neopterin and TNF-*α* and also significant but weak correlation between PTX3 at 24 h and level of neopterin and TNF-*α* in whole group of patients.

In this paper we demonstrate for the first time that increased levels of PTX3 in MI patients are significantly associated with higher levels of NO_*x*_, stable products of nitric oxide metabolism. The activity of inducible NO synthase is particularly interesting; the high levels of NO could lead to the worsening of left ventricular function, vasodilatation, and progression of hypotension [35].

Myocardial ischemia and subsequent reperfusion lead to increased production of reactive oxygen species (ROS) [36]. The toxic effects of ROS lead to damage to all cells components, including DNA, lipids, and proteins. Determination of individual ROS is very difficult, and their amount is assessed through appropriate markers. A marker of oxidative damage to DNA is 8-OHdG. In our study we found a weak significant correlation between levels of 8-OHdG upon hospital admission and PTX3 concentration 24 h after MI onset. Previously reported studies have shown that increased serum levels of 8-OHdG in patients with MI correspond with the severity of heart failure and left ventricular dysfunction [37, 38]. On the other hand, in case of MDA, another marker of oxidative stress, no significant correlation with levels of PTX3 was found.

We also evaluated the association between PTX3 and total antioxidant capacity of plasma, which was determined by the FRAP assay. We found a weak positive correlation between levels of PTX3 and FRAP. However, FRAP includes among others a considerable contribution of uric acid, a potent antioxidant, whose levels are elevated in MI [39]. The concentrations of uric acid within tertiles of PTX3 showed the same tendency as FRAP (Table 3). Contrary to our results, the relationship between PTX3 level and compounds with antioxidant activity was demonstrated by Hill and colleagues [40] who found that endothelial cells produced lower levels of PTX3 if incubated in vitro with addition of antioxidant substances (*N*-acetylcysteine, trolox, and idebenone).

The increase in the total antioxidant capacity of plasma may be caused by the release of intracellular antioxidants into the bloodstream as a result of the lysis of erythrocytes and epithelium after MI [41]. We found a weak significant correlation between levels of PTX3 and SOD. SOD is free radical-scavenging enzyme and is one of the first lines of “cellular defence” against oxidative injury. The increase in SOD level was presumably due to damage of myocardial tissue during MI [42]. We did not find a significant correlation between PTX3 level and antioxidant vitamins (A and E) and thiols.

There are limitations to this study. First, the study cohort could be considered relatively limited; on the other hand we evaluated an extensive list of new blood-based biomarkers. Second, the study was carried out at a single centre. Nevertheless, we studied a homogenous group of STEMI patients with a precisely documented number of unique invasive parameters (LVEDP, TIMI flow of infarct-related arteries) before and after primary PCI. Third, evaluation of PTX3 level assessed at the time of supposed peak value is interesting. However, we consider that assessment of some prognostic biomarkers 24 h after the onset of chest pain is, from a prognostic viewpoint, more practical and this approach would allow comparison of the results of different studies.

## 5. Conclusions

We demonstrated that PTX3 levels assessed 24 h after MI onset were strong and sensitive predictors of 30-day and 1-year mortality and could be used to identify high-risk patients requiring increased care. According to our results, prognostic value of PTX3 as a single biomarker is at least as good as currently used TIMI or GRACE risk scoring systems or other powerful predictors like BNP. Increased level of PTX3 is also an independent predictor of combined end-point of left ventricle dysfunction or mortality at one year.

## Figures and Tables

**Table 1:** Baseline clinical characteristics of patients according to PTX3 level at 24 h after the onset of myocardial infarction.

Characteristics	PTX3/24 h tertile			*P* value^*^
	First tertile	Second tertile	Third tertile	
	*n* = 87	*n* = 89	*n* = 86	
Age (years)	58.1 (49.0; 75.0)	63.7 (48.8; 79.7)	68.5 (53.0; 79.8)	**<0.001**
Female sex	17 (19.5)	30 (33.7)	30 (34.9)	**0.041**
Systolic BP (mmHg)	140 (105; 180)	140 (101; 190)	140 (85; 180)	0.411
Diastolic BP (mmHg)	80 (60; 105)	79 (55; 105)	80 (50; 97)	0.278
BMI	28.4 (23.8; 36.4)	27.7 (22.7; 35.1)	27.1 (21.1; 34.5)	0.096
Prior instability	33 (37.9)	27 (30.3)	23 (26.7)	0.273
Hypertension	53 (60.9)	52 (58.4)	53 (61.6)	0.902
Diabetes mellitus	17 (19.5)	19 (21.3)	32 (37.2)	**0.016**
Dyslipoproteinemia	80 (92.0)	69 (77.5)	71 (82.6)	0.023
History of MI	8 (9.2)	11 (12.4)	14 (16.3)	0.371
COPD	3 (3.4)	6 (6.7)	3 (3.5)	0.505
ACE inhibitors	19 (21.8)	26 (29.2)	20 (23.3)	0.490
Beta blockers	18 (20.7)	26 (29.2)	28 (32.6)	0.188
Statins	15 (17.2)	19 (21.3)	18 (20.9)	0.752
ARBs	9 (10.3)	3 (3.4)	9 (10.5)	0.105
Initial TIMI flow				0.055
0	41 (47.1)	58 (65.2)	53 (61.6)	
1	9 (10.3)	13 (14.6)	7 (8.1)	
2	18 (20.7)	7 (7.9)	13 (15.1)	
3	19 (21.8)	11 (12.4)	13 (15.1)	
Final TIMI flow				**0.018**
0	0 (0.0)	4 (4.5)	1 (1.2)	
1	0 (0.0)	0 (0.0)	2 (2.3)	
2	4 (4.6)	12 (13.5)	9 (10.5)	
3	83 (95.4)	73 (82.0)	74 (86.0)	
LVEDP (mmHg)	23 (11; 34)	26 (13; 38)	27 (13; 40)	**0.006**
LVEF (%)	55 (40; 68)	52 (34; 70)	45 (25; 62)	** <0.001**
Killip				**<0.001**
I	74 (85.1)	70 (78.7)	48 (55.8)	
II	12 (13.8)	18 (20.2)	23 (26.7)	
III + IV	1 (1.1)	1 (1.1)	15 (17.4)	
Acute heart failure	13 (14.9)	19 (21.3)	38 (44.2)	** <0.001**
Acute kidney injury	6 (6.9)	2 (2.2)	12 (14.0)	**0.011**
30day mortality	0 (0.0)	1 (1.1)	9 (10.5)	** <0.001**
1-year mortality	0 (0.0)	4 (4.5)	18 (20.9)	** <0.001**

All categorical variables represented as *n* (%) and continuous variables as median (the 5th; 95th percentiles).

^*^ *P* values from the Kruskal-Wallis test or *χ* ^2^ maximum-likelihood test (categorical variables).

BP: blood pressure; BMI: body mass index; MI: myocardial infarction; COPD: chronic obstructive pulmonary disease; ACE: angiotensin-converting enzyme; ARBs: antagonists for the type-2 receptor of angiotensin II; TIMI: thrombolysis in myocardial infarction; LVEDP: left ventricular end-diastolic pressure; LVEF: left ventricular ejection fraction; Killip: Killip classification of heart failure.

**Table 2:** Comparison of laboratory characteristics of patients divided according to PTX3 level in 24 h after the onset of chest pain.

Laboratory parameter^a^	PTX3/24 h tertiles			*P* value^*^
	First tertile	Second tertile	Third tertile	
	*n* = 87	*n* = 89	*n* = 86	
PTX3 (ng/mL)	1.0 (0.3; 1.4)	2.1 (1.6; 3.3)	6.4 (3.6; 45)	**<0.001**
Troponin I (pg/mL)	24.3 (1.0; 125)	56.3 (4.0; 150)	79.9 (4.8; 228)	**<0.001**
BNP (pg/mL)	191 (57.9; 581)	328 (89.0; 845)	490 (108.3; 1 485)	**<0.001**
NO_*x*_ (*μ*mo/L)	23.0 (12.0; 47.0)	24.0 (11.0; 59.0)	31.0 (20.0; 78.0)	**<0.001**
TNF-*α* (pg/mL)	2.1 (0.7; 6.4)	2.5 (0.9; 6.8)	3.1 (1.4; 8.3)	**0.009**
Neopterin (nmol/L)	6.4 (4.2; 12)	7.9 (4.9; 21)	9.5 (5.7; 27)	**<0.001**
MDA (*μ*mol/L)	0.5 (0.1; 1.0)	0.5 (0.3; 1.1)	0.5 (0.1; 0.9)	0.153
8-OHdG (ng/mL)	8.5 (4.7; 13)	7.6 (5.4; 12)	7.9 (4.7; 10)	0.311
SOD (ng/mL)	66 (26; 204)	64 (25; 226)	89 (36; 601)	**0.001**
FRAP (*μ*mol/L)	876 (520; 1 223)	818 (412; 1 197)	972 (572; 1 774)	**0.002**
Vitamin A (*μ*mol/L)	2.9 (1.5; 4.4)	2.6 (1.6; 4.6)	2.7 (1.8; 5.2)	0.283
Vitamin E (*μ*mol/L)	30.5 (17.2; 51.8)	30.7 (16.2; 43.8)	29.0 (18.0; 45.7)	0.670

Values are shown as the median (the 5th; 95th percentiles).

^*^ *P* value based on the Kruskal-Wallis test.

^a^Laboratory parameters were analyzed 24 h after the onset of chest pain.

PTX3: pentraxin-3; BNP: B-type natriuretic peptide; NO_*x*_: nitrite/nitrate; TNF-*α*: tumor necrosis factor alpha; MDA: malondialdehyde; 8-OHdG: 8-hydroxy-2′-deoxyguanosine; SOD: superoxide dismutase; FRAP: ferric-reducing ability of plasma.

**Table 3:** Laboratory parameters measured upon admission: the patients were divided according to PTX3 level in 24 h after the onset of chest pain.

Laboratory parameter^a^	PTX3/24 h tertiles			*P* value^*^
	First tertile	Second tertile	Third tertile	
	*n* = 87	*n* = 89	*n* = 86	
Leukocytes	11.7 (7.4; 18.1)	11.7 (6.6; 18.8)	12.9 (7.7; 22.4)	**0.043**
Glycaemia (mmol/L)	7.4 (5.3; 16)	8.4 (6.1; 13)	8.4 (5.8; 20)	**0.009**
Cholesterol (mmol/L)	5.5 (3.7; 7.3)	4.9 (3.5; 7.1)	5.2 (3.1; 7.2)	**0.015**
Triglycerides (mmol/L)	2.2 (0.8; 6.2)	1.6 (0.7; 3.7)	1.4 (0.5; 3.8)	**<0.001**
HDL (mmol/L)	1.2 (0.8; 1.7)	1.2 (0.8; 2.1)	1.5 (0.6; 2.0)	**<0.001**
LDL (mmol/L)	3.2 (1.7; 4.8)	2.7 (1.6; 5.1)	3.1 (1.2; 4.6)	**0.024**
Creatinine (*μ*mol/L)	83 (59; 119)	87 (60; 138)	90 (62; 151)	**0.033**
Uric acid (*μ*mol/L)	325 (206; 475)	303 (180; 488)	359 (198; 642)	**0.013**
PTX3 (ng/mL)	0.6 (0.2; 2.0)	1.0 (0.4; 3.0)	1.5 (0.3; 7.9)	**<0.001**
BNP (pg/mL)	46.1 (15.0; 179)	81.8 (15.3; 489)	106.3 (15.1; 605)	**<0.001**
TNF-*α* (pg/mL)	1.4 (1.1; 3.7)	3.3 (1.9; 4.1)	2.9 (1.7; 12)	0.399
MDA (*μ*mol/L)	0.6 (0.3; 1.4)	0.5 (0.3; 1.1)	0.6 (0.2; 1.2)	0.462
8-OHdG (ng/mL)	7.1 (4.5; 9.5)	7.3 (4.5; 11)	8.3 (5.2; 14)	**<0.001**
FRAP (*μ*mol/L)	910 (645; 1 309)	828 (467; 1 330)	987 (493; 1 589)	0.052
Cysteine (*μ*mol/L)	260 (192; 326)	259 (203; 348)	263 (163; 391)	0.924
Homocysteine (*μ*mol/L)	9.5 (5.6; 16)	10.6 (5.5; 18)	9.9 (4.0; 20)	0.539
Cysteinylglycine (*μ*mol/L)	41.5 (30.5; 62.1)	40.2 (29.3; 59.8)	41.1 (22.3; 58.2)	0.182
Glutathione (*μ*mol/L)	2.2 (1.1; 3.3)	2.3 (1.3; 3.3)	2.1 (1.0; 3.7)	0.402

Values are shown as the median (the 5th; 95th percentiles).

^*^ *P* value based on the Kruskal-Wallis test.

^a^Laboratory parameters were analyzed upon hospital admission.

HDL: high density lipoprotein; LDL: low density lipoprotein; PTX3: pentraxin-3; BNP: B-type natriuretic peptide; TNF-*α*: tumor necrosis factor alpha; MDA: malondialdehyde; 8-OHdG: 8-hydroxy-2′-deoxyguanosine; FRAP: ferric-reducing ability of plasma.

**Table 4:** PTX3 levels in selected groups of patients.

	PTX3 (ng/mL)	*P* value
Gender		
Male	2.05 (0.49; 25.7)	**0.031**
Female	2.52 (0.53; 19.4)	
Initial TIMI flow		
0	2.28 (0.66; 36.0)	0.061
1	1.91 (0.58; 15.3)	
2	1.67 (0.41; 31.7)	
3	1.58 (0.53; 6.83)	
Final TIMI flow		
0 + 1	3.35 (1.96; 77.2)	0.067
2	2.71 (0.99; 36.0)	
3	2.07 (0.49; 19.4)	
Killip		
I	1.97 (0.44; 8.53)	**<0.001**
II	2.80 (0.77; 31.7)	
III + IV	14.5 (0.93; 125)	
Acute heart failure		
no	1.97 (0.44; 8.53)	**<0.001**
yes	4.04 (0.81; 45.1)	
Acute kidney injury		
no	2.10 (0.49; 15.1)	**0.015**
yes	3.83 (0.87; 77.2)	
Diabetes mellitus		
no	2.02 (0.49; 14.5)	**0.003**
yes	2.89 (0.63; 45.2)	
Hypercholesterolemia		
no	2.75 (0.82; 37.5)	**0.021**
yes	2.05 (0.49; 15.3)	

Values are shown as median (the 5th; 95th percentiles).

^*^ *P* values from the Kruskal-Wallis test or *χ* ^2^ maximum-likelihood test.

TIMI: thrombolysis in myocardial infarction; Killip: Killip classification of heart failure.

**Table 5:** Relationship between PTX3 level at 24 h after the onset of chest pain and selected patient characteristics.

	Spearman correlation coefficient	*P* value^*^
Age	0.371	**<0.001**
BMI	−0.139	**0.024**
Systolic BP	−0.092	0.139
Diastolic BP	−0.087	0.158
LVEDP	0.188	**0.003**
LVEF	−0.328	**<0.001**
Glycaemia (admission)	0.244	**<0.001**
Creatinine (admission)	0.188	**0.002**
Troponin I (max)	0.376	**<0.001**
BNP (admission)	0.338	**<0.001**
BNP	0.498	**<0.001**
NO_*x*_	0.329	**<0.001**
TNF-*α*	0.271	**0.002**
Neopterin	0.448	**<0.001**
MDA	−0.032	0.617
8-OHdG (admission)	0.275	**<0.001**
8-OHdG	−0.170	0.329
SOD	0.182	**0.006**
FRAP	0.172	**0.006**
Vitamin A	−0.067	0.346
Vitamin E	−0.089	0.211

Laboratory parameters were analyzed 24 h after the onset of chest pain or upon hospital admission.

BMI: body mass index; BP: blood pressure; LVEDP: left ventricular end-diastolic pressure; LVEF: left ventricular ejection fraction; BNP: B-type natriuretic peptide; NO_*x*_: nitrite/nitrate; TNF-*α*: tumor necrosis factor alpha; MDA: malondialdehyde; 8-OHdG: 8-hydroxy-2′-deoxyguanosine; SOD: superoxide dismutase; FRAP: ferric-reducing ability of plasma.

**Table 6:** ROC analyses for prediction of 30-day and 1-year mortalities according to the TIMI and GRACE risk scores and levels of PTX3 at 24 h after the onset of myocardial infarction and upon admission.

Marker	AUC (95% CI)	*P* value	AUC (95% CI)	*P* value
	30-day mortality		1-year mortality	
TIMI	0.795 (0.741–0.842)	**<0.001**	0.691 (0.582–0.800)	**<0.001**
GRACE	0.841 (0.717–0.966)	**<0.001**	0.783 (0.672–0.893)	**<0.001**
PTX3	0.873 (0.801–0.945)	**<0.001**	0.835 (0.769–0.900)	**<0.001**
BNP	0.812 (0.684–0.941)	**<0.001**	0.812 (0.715–0.909)	**<0.001**
TIMI + PTX3	0.847 (0.797–0.888)	**<0.001**	0.761 (0.669–0.853)	**<0.001**
GRACE + PTX3	0.851 (0.730–0.972)	**<0.001**	0.791 (0.682–0.901)	**<0.001**
PTX3/adm^*^	0.652 (0.459–0.846)	0.143	0.663 (0.544–0.782)	**0.016**
BNP/adm^*^	0.664 (0.500–0.828)	0.078	0.727 (0.605–0.848)	**<0.001**
TIMI + PTX3/adm^*^	0.863 (0.815–0.903)	**<0.001**	0.760 (0.656–0.864)	**<0.001**
GRACE + PTX3/adm^*^	0.891 (0.759–1.000)	**<0.001**	0.833 (0.728–0.939)	**<0.001**

^∗^PTX3 or BNP at time of admission.

AUC: area under curve; CI: confidence interval; TIMI: thrombolysis in myocardial infarction (risk score); GRACE: global registry of acute coronary events (risk score); PTX3: pentraxin-3 at 24 h after onset of myocardial infarction; BNP: B-type natriuretic peptide at 24 h after onset of myocardial infarction.

**Table 7:** Predictive power and optimal cut-offs of biomarkers for the prediction of combined endpoint (mortality or EF ≤40% at one year).

Predictors	AUC (95% CI)	*P* value	Cut-off^*^	Sensitivity	Specificity
PTX3	0.756 (0.675; 0.837)	<0.001	>2.1	0.864	0.572
Troponin I (max)	0.705 (0.610; 0.801)	<0.001	>90	0.568	0.826
BNP	0.800 (0.731; 0.870)	<0.001	>500	0.636	0.827

Results are based on ROC analysis.

^*^Values in ng/mL (PTX3) and pg/mL (troponin I, BNP).

AUC: area under curve; CI: confidence interval; PTX3: pentraxin-3 at 24 h after onset of myocardial infarction; BNP: B-type natriuretic peptide at 24 h after onset of myocardial infarction.

**Table 8:** Predictors of combined end-point (mortality or EF <40% at one year).

Predictors	Univariate	*P* value^*^	Multivariate without PTX3	*P* value^*^	Multivariate with PTX3	*P* value^*^
	OR (95% CI)		OR (95% CI)		OR (95% CI)	
PTX3 >2.1 ng/mL	8.304 (3.364; 20.498)	**<0.001**			3.700 (1.332; 10.279)	**0.012**
Troponin I (max) >90 pg/mL	6.241 (3.102; 12.556)	**<0.001**	4.329 (1.966; 9.531)	**<0.001**	3.321 (1.461; 7.549)	**0.004**
BNP >500 pg/mL	8.361 (4.104; 17.033)	**<0.001**	6.777 (3.068; 14.970)	**<0.001**	4.990 (2.196; 11.340)	**<0.001**

Women	1.125 (0.558; 2.267)	0.743				
Age	1.035 (0.998; 1.072)	0.063				
BMI	1.057 (0.980; 1.140)	0.151				
Systolic BP	0.991 (0.979; 1.003)	0.158				
Heart rate	1.027 (1.008; 1.046)	**0.005**	1.029 (1.007; 1.052)	**0.009**	1.030 (1.008; 1.054)	**0.008**
Glycaemia at entry	1.124 (1.044; 1.211)	**0.002**				
Creatinine at entry	1.024 (1.011; 1.037)	**<0.001**				
Diabetes mellitus	3.019 (1.531; 5.952)	**0.001**				
Hypertension	2.644 (1.241; 5.634)	**0.012**				
Dyslipoproteinemia	1.033 (0.425; 2.509)	0.943				
History of MI/PCI/CABG	2.745 (1.253; 6.013)	**0.012**				
Pain onset-balloon time	0.994 (0.976; 1.013)	0.543				
IRA-LAD/left main	2.090 (1.079; 4.050)	**0.029**				
Three vessels diseases	2.183 (1.408; 3.383)	**<0.001**	2.079 (1.230; 3.514)	**0.006**	1.985 (1.159; 3.398)	**0.012**

Reclassification					Rec. value (95% CI)	*P* value^a^

cfNRI IDI	Reclassification analysis computed for multivariate model containing PTX3 against model without PTX3				0.882 (0.637; 1.126) 0.028 (0.006; 0.051)	**<0.001** **0.014**

^∗^Based on logistic regression.

^a^Based on reclassification analysis (cfNRI, IDI).

PTX3: pentraxin-3 at 24 h after onset of myocardial infarction; BNP: B-type natriuretic peptide at 24 h after onset of myocardial infarction; BMI: body mass index; BP: blood pressure; MI: myocardial infarction; PCI: percutaneous coronary intervention; CABG: coronary artery bypass grafting; IRA: infarct related artery; LAD: left anterior descending.

## Abbreviations

AHF: Acute heart failure
AUC: Area under the curve
BNP: B-type natriuretic peptide
cfNRI: Category-free net reclassification improvement
CRP: C-reactive protein
FRAP: Ferric-reducing ability of plasma
GRACE: Global registry of acute coronary events
8-OhdG: 8-Hydroxy-2′-deoxyguanosine
IDI: Integrated discrimination improvement
LVEDP: Left ventricular end-diastolic pressure
LVEDV: Left ventricular end-diastolic volume
LVEF: Left ventricular ejection fraction
LVESV: Left ventricular end-systolic volume
MDA: Malondialdehyde
MI: Myocardial infarction
p38MAPK: p38 mitogen-activated protein kinase
NO_*x*_: Nitrite/nitrate
NO: Nitric oxide
NRI: Net reclassification improvement
NSTEMI: Non-ST-elevation myocardial infarction
PCI: Percutaneous coronary intervention
PTX3: Pentraxin-3
ROS: Reactive oxygen species
SOD: Superoxide dismutase
STEMI: ST-elevation myocardial infarction
TIMI: Thrombolysis in myocardial infarction
TNF-*α*: Tumor necrosis factor alpha.
